# Whole mitochondrial genome analyses of Han population from Shandong of China using massively parallel sequencing

**DOI:** 10.3389/fgene.2024.1513412

**Published:** 2024-11-29

**Authors:** Jiashuo Zhang, XueBo Li, Anqi Chen, Mingxia Ding, Liangliang Li, Yinghua Qi, Chunli Ding, Dawei Cai, Suhua Zhang

**Affiliations:** ^1^ School of Archaeology, Jilin University, Changchun, Jilin, China; ^2^ Key Laboratory of Evidence Identification in Universities of Shandong Province, Shandong University of Political Science and Law, Jinan, Shandong, China; ^3^ Institute of Forensic Science, Shandong University of Political Science and Law, Jinan, Shandong, China; ^4^ Institute of Forensic Science, Fudan University, Shanghai, China; ^5^ Obstetrics and Gynecology Department, Second Hospital of Shandong University, Jinan, Shandong, China

**Keywords:** forensic genetics, whole mitochondrial genome, Shandong Han, massively parallel sequencing, population genetics

## Abstract

**Introduction:**

Mitochondrial DNA (mtDNA) has been extensively utilized in archeology, human evolutionary genetics, and forensic genetic for over three decades, primarily due to its maternal inheritance and relatively high mutation rate. The Chinese Han, the largest and most widely distributed ethnic group in China, have been the focus of numerous genetic studies. However, the forensic parameters and genetic structure of the Shandong Han, specifically in relation to the whole mitochondrial genome, remain undocumented.

**Methods:**

We performed whole mitochondrial genome sequencing on 141 unrelated Han individuals from Shandong province using massively parallel sequencing.

**Results:**

A total of 135 unique mtDNA haplotypes were identified, classified into 105 haplogroups, resulting in a haplotype diversity value of 0.9993. The discriminatory capacity of whole mitochondrial genome was calculated at 0.9574, compared to 0.8936 when only the control region was analyzed. The majority of the haplogroups observed were specific to East Asian lineages, including D4, D5 and F1. Population comparisons revealed that the modern Shandong Han share genetic connections with ancient populations from the Yellow River and West Liao River basins. Additionally, the Shandong Han may have integrated a significant number of maternal lineages from other regions during their development. The demographic expansion of the Shandong Han is estimated to have occurred approximately 9,000 years ago, corresponding to the Neolithic period, a time of significant cultural and technological development.

**Discussion:**

The dataset generated in this study is available in the EMPOP database under the accession number EMP00886 and will serve as an important mtDNA reference for forensic casework in China. The study of whole mitochondrial genome based on the analysis of matrilineal genetic structure of the Shandong Han population can help to enrich the forensic mtDNA reference database in East Asia and provide reference for future archeology and forensic genetics research.

## 1 Introduction

Human mitochondrial DNA (mtDNA) possesses several unique characteristics, such as high copy number per cell, absence of recombination, rapid mutational rate, and maternal inheritance. These features make mtDNA a powerful tool across a variety of fields, including medical genetics, anthropology, population genetics, archeology, and forensic genetics (Derenko et al., 2007; Murphy, 2018; Simão et al., 2018; Wang et al., 2020; Ning et al., 2021; Lintao et al., 2024; Zheng et al., 2024). Its value is especially evident in ancient DNA analysis and forensic applications, where DNA samples are often highly fragmented or degraded, such as in bones, nails and hair shafts without roots (Irfan et al., 2024; Li et al., 2024), which may lack sufficient nuclear DNA. Due to its robustness, mtDNA is commonly used when nuclear DNA is either unavailable or inadequate for analysis.

Historically, researches have focused on sequencing the hypervariable regions Ⅰ, Ⅱ and Ⅲ (HV Ⅰ, HV Ⅱ and HV Ⅲ) of the non-coding control region (CR), along with a selection of specific single nucleotide polymorphisms (SNPs) from the coding region (CodR) (Hong et al., 2015; Chaitanya et al., 2016). However, the CR alone offers limited polymorphism data, which can reduce the effectiveness of mtDNA analysis in forensic casework. Studies have shown that more than 70% of mtDNA variants are located outside the hypervariable regions, highlighting the advantage of sequencing the entire mitochondrial genome for greater discrimination and precise haplogroup classification (Brotherton et al., 2013; Zhou et al., 2016).

Traditionally, mtDNA analysis relied on Sanger sequencing, a time-consuming and inefficiency method, making large-scale mtDNA sequencing projects impractical (Ma et al., 2018). Massively parallel sequencing (MPS), by contrast, offers a more efficient approach, yielding higher throughput data with increased resolution. This technological advancement allows for the creation of larger and more detailed mtDNA databases, significantly enhancing genetic research (King et al., 2014). The EMPOP database (https://empop.online/), which now includes 48,572 quality-controlled mitotypes, has benefited substantially from MPS-based mtDNA sequencing. Among these, 46,963 mitotypes cover HVS-I and HVS-II, 38,361 span the entire CR, and 4,289 represent complete mitochondrial genomes.

The Han Chinese population, the largest ethnic group in the world with a population of approximately 1.4 billion, has been of great interest to researchers in fields such as anthropology, archaeology, and forensic genetics (Liu et al., 2021). As the dominant ethnic group in China and Singapore (Chen et al., 2019), understanding the origins, migration patterns, and genetic relationships of the Han people is crucial for the study of East Asian populations. Shandong province, in particular, plays a significant historical and cultural importance, especially as the birthplace of Confucianism (Rong and Bahauddin, 2023). According to the seventh national population census, Han Chinese account for 99.11% of Shandong’s population. Despite this, previous studies on the Shandong Han population, particularly from the perspective of maternal inheritance, have been limited in both sample size and scope, often focusing only on HV I, HV II, and CR data (Yao et al., 2002b).

Moreover, ancient DNA studies suggest that ancient Shandong people genetically related to both Northern and Southern East Asian populations (Liu et al., 2021). Therefore, a comprehensive analysis of the complete mitochondrial genome would provide a deeper understanding of the genetic diversity and population dynamics in the region.

In this study, we sequenced the complete mitochondrial genomes of 141 healthy, unrelated Han individuals from Shandong using MPS technology. We performed a detailed analysis of haplogroup distribution, genetic diversity, point heteroplasmy, and maternal genetic structure within the Shandong Han population. Additionally, to further explore the genetic relationships between the Shandong Han and populations across Asia and Europe, we conducted principal component analyses (PCA) and network analyses. This research aims to enhance our understanding of the genetic makeup of the Shandong Han and provide valuable insights for future studies in population genetics, archaeology, and forensic science.

## 2 Materials and methods

### 2.1 Sample preparation and ethical statement

Saliva samples were collected from 141 unrelated Han individuals (79 males and 62 females) in Shandong province, China. Written informed consent was obtained from each participant. This study was approved by the Ethics Committee of the Scientific Research Institute at the Second Hospital of Shandong University (approval number: KYLL-2020(LW)-055) and adhered to the ethical guidelines of the world Medical Association (Association, 2013). All procedures were conducted in accordance with the principles of Declaration of Helsinki.

### 2.2 DNA extraction, library construction and sequencing

Genomic DNA was extracted from the saliva samples using the QIAamp DNA Mini Kit (QIAGEN, Germany) according to the manufacturer’s instructions. The concentration of gDNA was measured using the Invitrogen Qubit 4 Fluorometer (Thermo Fisher Scientific, United States). For downstream applications, the gDNA was normalized to 5 ng/μL and stored at −20°C until amplification.

DNA library construction was conducted using the MultipSeq™ AimumiCap Panel (Enlighten biotechnology company, China), which utilizes 129 paired primers for the PCR amplification of the entire mitochondrial genome. A non-template library negative control and a library positive control were introduced during library preparation. The multiplex PCR amplification was carried out in a 30 μL reaction mixture containing 1 μL of template DNA (5 ng/μL), 5 μL of RealCapChrMT Mix, 10 μL of 3×Enzyme HF and 14 μL of nuclease-free water. The PCR cycling conditions were as follows: an initial denaturation at 98°C for 3 min; 13 cycles of 98°C for 20 s and 58°C for 4 min; followed by 7 cycles of 98°C for 20 s and 72°C for 1 min; and a final extension at 72°C for 2 min. The amplified products were purified using Agencourt AMPure XP beads (Beckman Coulter, United States).

A second round of PCR amplification was carried out to add adapters and indexes. This reaction volume, with a total volume of 30 μL, included 10 μL 3×Enzyme HF, 18 μL of purified PCR products, 1 μL of I5 index, and 1 μL of I7 index. The thermal cycling reaction were: 98°C for 2 min; 6 cycles of 98°C for 15 s, 58°C for 15 s, and 72°C for 15 s; followed by a final extension at 72°C for 2 min.

After quantification, the libraries were sequenced using paired-end sequencing on the Illumina HiSeq X Ten platform. All mtDNA sequencing and subsequent data analysis were conducted according to the standards set by the International Society of Forensic Genetics (ISFG) and the U.S. Scientific Working Group on DNA Methods (SWGDAM) (Methods, 2013; Parson et al., 2014; Connell et al., 2022).

### 2.3 Sequencing data analyses

Redundant primers and indexes were removed using the Cutadapt software (https://github.com/marcelm/cutadapt/), and low-quality reads were filtered using Trimmomatic v0.39 (https://github.com/usadellab/Trimmomatic). The cleaned data were then aligned to the revised Cambridge Reference Sequence plus 64 bp using the BWA alignment tool. To minimize the potential for false positives caused by nuclear mitochondrial DNA (NUMTs) contamination, the sequences were also compared with the human reference genome hg19.

Reads successfully mapped to hg19 were extracted with Bedtools and realigned to rCRS, generating updated BAM files using Bowtie2 (Langmead and Salzberg, 2012). Mutation sites were identified, and variant data were exported in VCF format using GATK, Angsd, and Mia software (McKenna et al., 2010; Schönberg et al., 2011; Korneliussen et al., 2014). The final consensus sequence in FASTA format was generated with the Consensus.py script (https://github.com/TaizoAyase/consensus_creator).

### 2.4 MtDNA haplogroup assignment

The haplogroups of whole mtDNA sequences from the Shandong Han population were identified using HaploGrep3 (https://haplogrep.i-med.ac.at/) based on PhyloTree build 17 (Van Oven and Kayser, 2009). To ensure accuracy, the haplogroups were further validated using the SAM2 tool (Huber et al., 2018) integrated into EMPOP (Parson and Dür, 2007).

The EMPOP tools “EMPcheck” and “network” were adopted to identify and correct potential errors in the dataset. The finalized sequence data were submitted to EMPOP, and only quality-controlled mtDNA sequences were retained for subsequent population comparison analyses.

### 2.5 Statistical analyses

Haplogroup and haplotype frequencies in this study were derived from whole mitochondrial sequences and calculated using the direct counting method. The haplogtype match probability (HMP) was defined as $HMP=\sum p_{i}^{2}$ , where *pi* is represents the frequency of the *i-*th haplotype. Haplotype diversity (HD) was calculated using the formula $HD=n\left(1-\sum p_{i}^{2}\right)/\left(n-1\right)$ , where *n* is the sample size and *pi* is represents the frequency of the *i-*th haplotype (Purps et al., 2014).

The discrimination capacity (DC) was defined as the ratio between the number of distinct haplotypes and the total number of haplotypes (Purps et al., 2014). The nucleotide diversity (π), the number of segregating sites (S), neutrality tests (Tajima’s D and Fu’s Fs tests), and the average number of pairwise nucleotide differences (K) were estimated using DnaSP v6 based on the whole mitochondrial genomes (Rozas et al., 2017).

### 2.6 Population comparisons

To investigate the genetic relationships between the Shandong Han population and other global populations, we obtained 1,514 complete mitochondrial sequences from 15 populations across East Asia, South Asia, and Europe through the 1000 Genomes Project. Additionally, 188 mitochondrial genomes from five populations across North and West Asia were collected from the Human Genome Diversity Project (HGDP). A further 16,375 mtDNA sequences were collected from 26 provinces across China, along with 36 ancient mitochondrial genomes. Detailed information on all reference populations is provided in Supplementary Table S3.

The haplogroup of each reference mitochondrial genome (mitogenome) was identified using the HaploGrep3 (https://haplogrep.i-med.ac.at/). All reference FASTA files were aligned with MAFFT (https://mafft.cbrc.jp/alignment/software/) and merged into our dataset (Rozewicki et al., 2019). The PCA was performed on haplogroups frequencies using the FactoMineR v2.11 in R software (https://cran.r-project.org/web/packages/FactoMineR/index.html).

To further examine specific mtDNA haplogroups, a network analysis was conducted using the median-joining method in Popart (https://popart.maths.otago.ac.nz/) (Leigh et al., 2015). A Bayesian skyline plot (BSP) was generated with BEAST2 2.7.0 to infer the demographic history of the Shandong Han population applying the TN93 (Chen et al., 2020). The molecular clock was calibrated using the mutation rate defined by Soares et al. (2009). Tracer v1.7 (https://github.com/beast-dev/tracer/releases/tag/v1.7.2) was used to assess the convergence of the runs, ensuring effective sampling size (ESS) and to reconstruct the population dynamics over time (Rambaut et al., 2018).

## 3 Results and discussion

### 3.1 Quality control

The whole mitochondrial genomes generated in this study were carefully reviewed by two independent scientists. Consistent mtDNA haplotypes were then submitted to the EMPOP database (https://empop.online/). A total of 141 mtDNA haplotypes (listed in Supplementary Table S1) were validated and approved by EMPOP colleagues and are now accessible through the EMPOP browser under accession number EMP00886.

The average number of mapped reads per individual was 505,209 ± 171,486, with an overall mean read depth of 3,721× ± 1,241× per individual (as illustrated Supplementary Figure S1). In general, higher sequencing depth increases the confidence in calling a variant at a specific location. As shown in Supplementary Figure S1, the average read depth for all individuals ranged from 445× to 7,615×, suggesting a considerable degree of reliability. This robust coverage suggests that the MultipSeq™ AimumiCap panel kit from Enlighten Biotechnology Company (Shanghai, China) performed well in capturing complete mitochondrial genomes, demonstrating its effectiveness for comprehensive mitogenome sequencing. The consistency of the read depth across the samples underscores the reliability of the kit for high-throughput genetic analysis.

### 3.2 MtDNA haplogroup distribution

**FIGURE 1:**
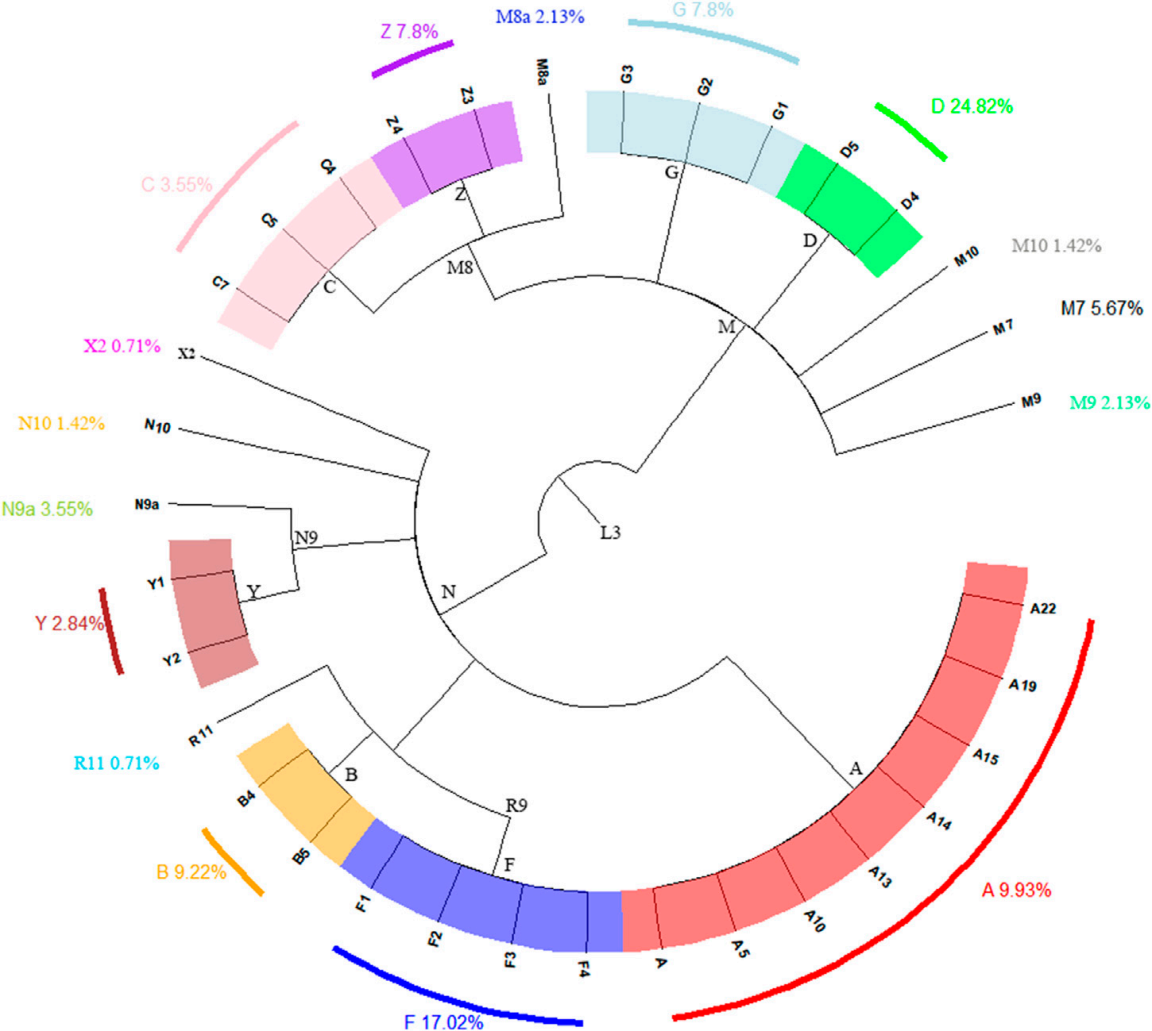
The phylogenetic relationship of coarse mtDNA haplogroups in this study and their clan-based frequencies among the Shandong Han population.

A total of 105 haplogroups and 135 haplotypes were identified from the 141 complete mitochondrial genomes of the Shandong Han population, as shown in Supplementary Table S1. These haplogroups were determined using HaploGrep3 based on PhyloTree build 17 and verified manually. The matrilineal ancestry of the Shandong Han population was predominantly composed of East Asian-specific lineages (99.29%), with a small presence of the European-specific haplogroups X2 (0.71%) (see Figure 1) (Kivisild et al., 2002; Kong et al., 2003; Reidla et al., 2003). This strong representation of East Asian lineages reflects the population’s genetic heritage, while the minor presence of haplogroup X2 points to limited genetic input from Europe.

The East Asian-specific lineages were primarily distributed across various sub-haplogroups of M, including D (24.82%), G (7.8%), Z (7.8%), and M7 (5.67%), with an additional 1.42% classified as M10. Other significant haplogroups included A (9.93%), B (9.22%), F (17.02%), Y (2.84%), N9a (3.55%), and N10 (1.42%), all of which fall under the N sub-haplogroup. Among these, haplogroup D was the most prevalent, followed by F, A, B, Z, and G.

Haplogroup D, the largest group in the Shandong Han population, consisted of two sister subclades: D4 (17.73%) and D5 (7.09%). Subclade D4, particularly D4j, is highly common in Northern and Northeastern China (Yao et al., 2019), while D5 is widely distributed among Han Chinese populations (Yao et al., 2002b). The most frequent haplogroup D4 subclade in Shandong Han was D4j, followed by D4a, which is common in Han populations from North and Northeast China (Li et al., 2019). Haplogroup D5 includes D5a (2.84%), D5b (2.84%), and D5c (1.42%), found predominantly in East and Southeast Asia (Peng et al., 2011). The discovery of haplogroups D4 and D5 in ancient Shandong populations, dating back approximately 9,500-4,600 years, indicates that early Shandong populations shared the matrilineal genetic characteristics of northern East Asian populations (Liu et al., 2021). The suggests that these haplogroups have been stable over time in the Shandong Han.

Haplogroup F, the second-largest group, was mainly represented by subclade F1 (10.64%), followed by F2 (4.96%), with smaller proportions of F3 (0.71%) and F4 (0.71%). F1 is widespread in Northern China, while F2 is more common in Southern and Southwestern China (Li et al., 2019). Haplogroup A (9.93%) is predominantly found in Northern and Eastern Asia (Derenko et al., 2007). Within haplogroup B (9.22%), B4 (3.55%) and B5 (5.67%) were prevalent, both commonly seen in mainland Southeast Asia and Southern China (Derenko et al., 2012; Duong et al., 2018).

Haplogroup G (7.8%) was divided into G1 (1.42%), G2 (4.26%), and G3 (2.13%), with these subclades typically found among Japanese and Mongolic-speaking populations (Yao et al., 2002a). Haplogroup Z (7.8%) was further classified into Z3 (4.26%) and Z4 (3.55%), which are characteristic of East Eurasian populations, particularly in Northern China and Central Asia (Derenko et al., 2007).

In addition to the major haplogroups, several other haplogroups were identified at lower frequencies, including M7, M8a, M9, M10, N9a, N10, and R11. Sub-haplogroup M8a, a branch of the broader M8 haplogroup, is prevalent in Central and Northern China (Yao et al., 2002b), while M7 is typically found in Southeast Asian and Southern Chinese populations (Peng et al., 2011). Haplogroup R11 is widely distributed across East Asia, including Japan, Korea, and Southern China (Kong et al., 2003). Notably, the European-specific haplogroup X2 was also detected at a low frequency in the Shandong Han population, highlighting the region’s minor yet significant genetic ties to Europe.

These findings provide a comprehensive view of the matrilineal genetic diversity within the Shandong Han population, emphasizing the predominance of East Asian lineages and the presence of distinct haplogroups with varied geographic distributions.

### 3.3 MtDNA genetic diversity and heteroplasmy

It is note that the haplotype F1a1c was observed in three individuals and four haplotypes (F1a1, M9a1a1c1a, D4j15 and D4) were shared between two individuals each. The value of HD was calculated at 0.9993, with a DC of 0.9574. The value of HMP was determined to be 0.0078. Several genetic diversity metrics were also calculated, including the number of polymorphic (segregating) sites (535), nucleotide diversity (0.0019 ± 0.0004), and the average number of pairwise differences (31.418 ± 1.1291), all of which provide insights into the effective population size of the Shandong Han.

Neutrality tests, such as Tajima’ D (−2.2369) and Fu’s Fs (−32.7860), yielded significantly negative results, indicating potential recent population expansions or evidence of positive selection in the Shandong Han. Table 1 presents a summary of statistics for the CR, CodR, and whole mtDNA sequence data. Compared to the CR data alone, the whole mtDNA sequence analysis demonstrated a 15.22% reduction in HMP, while the number of unique haplogroups, unique haplotypes, and haplotype diversity increased by 15.24%, 6.67%, 0.15%, respectively. Moreover, the discriminatory capacity increased from 0.8936 with CR haplotypes to 0.9574 with the inclusion of whole mtDNA sequences in the Shandong Han samples.

**TABLE 1:** Summary statistics for whole mtDNA sequence data from 141 individuals of Shandong Han population.

Parameters	Control region (CR)	Coding region (CodR)	Whole mtDNA	Percentage increase of CR to whole mtDNA
Variants	1,500	3,554	5,054	70.32%
Unique haplotypes	126	121	135	6.67%
Unique haplogroups	89	101	105	15.24%
Haplotype diversity	0.9978	0.9972	0.9993	0.15%
Haplotype match probability	0.0092	0.0099	0.0078	−15.22%
Discrimination capacity	0.8936	0.8582	0.9574	6.66%

These findings, in combination with prior research, suggest that the lack of mutations in the CR may hinder precise haplogroup classification (Wang et al., 2022). Overall, the results indicate that the whole mtDNA sequence data exhibits a high degree of discriminatory power and is a valuable tool for studying maternal lineage in the Shandong Han population. Additionally, it enhances the retrieval of genetic data and underscores the significance of whole mtDNA sequencing in forensic genetics.

In this study, we applied a detection threshold for point heteroplasmy at a minor allele frequency (MAF) of ≧15% (Just et al., 2015; Zhou et al., 2016). A total of 16 potential point heteroplasmy positions (PHP) were identified in 16 samples (Supplementary Table S2). Four potential PHP positions were excluded according to the following criteria: (1) PHP at position 574 (5/141) in the CR and positions 1,393 (3/141) and 1,405 (5/141) in the CodR were deemed uncommon within a single population, and discrepancies were observed between EMPOP and IGV at these positions; (2) the position 8,701 did not meet the double-strand confirmation requirement, as 8701G was only observed on the forward strands.

Our results demonstrate that PHP positions were randomly distributed across the mtDNA, with rare occurrences of PHPs, consistent with previous studies by Wang et al. (2020). In general, PHPs show significant potential for applications, particularly for individual identification, distinguishing close maternal relatives, and determining age, tissue type, or environmental factors (Just et al., 2015).

### 3.4 Genetic relationship of the Shandong Han with other populations

**FIGURE 2:**
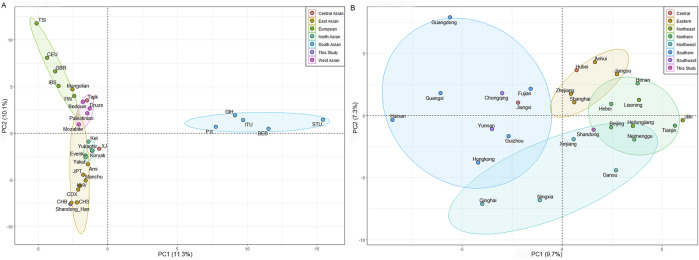
**(A)** Principal component analysis (PCA) plot showing the genetic relationships between Shandong Han and Eurasia populations based on the haplogroup frequencies. **(B)** A PCA plot showing the genetic relationships between Shandong Han and other Han groups from different regions of China. PC1 and PC2 are derived from the total variance.

To explore the genetic relationships between the Shandong Han population and Eurasia populations, PCAs were performed using haplogroup frequencies from Eurasia and China datasets (Supplementary Table S3). As illustrated in Figure 2, the PCA of Eurasia populations revealed five genetically distinct cluster: European, East Asian, North Asian, South Asian, and West Asian. The first two components explain 21.4% of the total variance (PC1: 11.3%, PC2: 10.1%). PC1 differentiated South Asian populations from the other reference populations, while PC2 primarily separated Europe populations from the rest. The Shandong Han population was located within the broader East Asian cluster, closely aligned with the Beijing Han (CHB) and Southern Han (CHS) groups (Figure 2A), indicating a strong genetic connection between these populations.

To further investigate the genetic structure of the Shandong Han and examine population substructure among different Han groups, a separate PCA was conducted using only Chinese Han data. The first two components accounted for 17% of the genetic variation (PC1: 9.7% and PC2: 7.3%). PC1 distinguished Northern China Han populations from Southern China Han populations, while PC2 separated Eastern Han groups from those in Northwest China (Figure 2B). The positioning of the Shandong Han, Beijing Han and Hebei Han populations in the PCA closely matched their geographic locations, suggesting that their maternal genetic composition reflects historical migration patterns and genetic contributions from various groups during the development of the Han population in China.

These results provide insights into the genetic diversity within the Han population, as well as the broader connections between East Asian populations and their neighboring regions in Asia and Europe.

**FIGURE 3:**
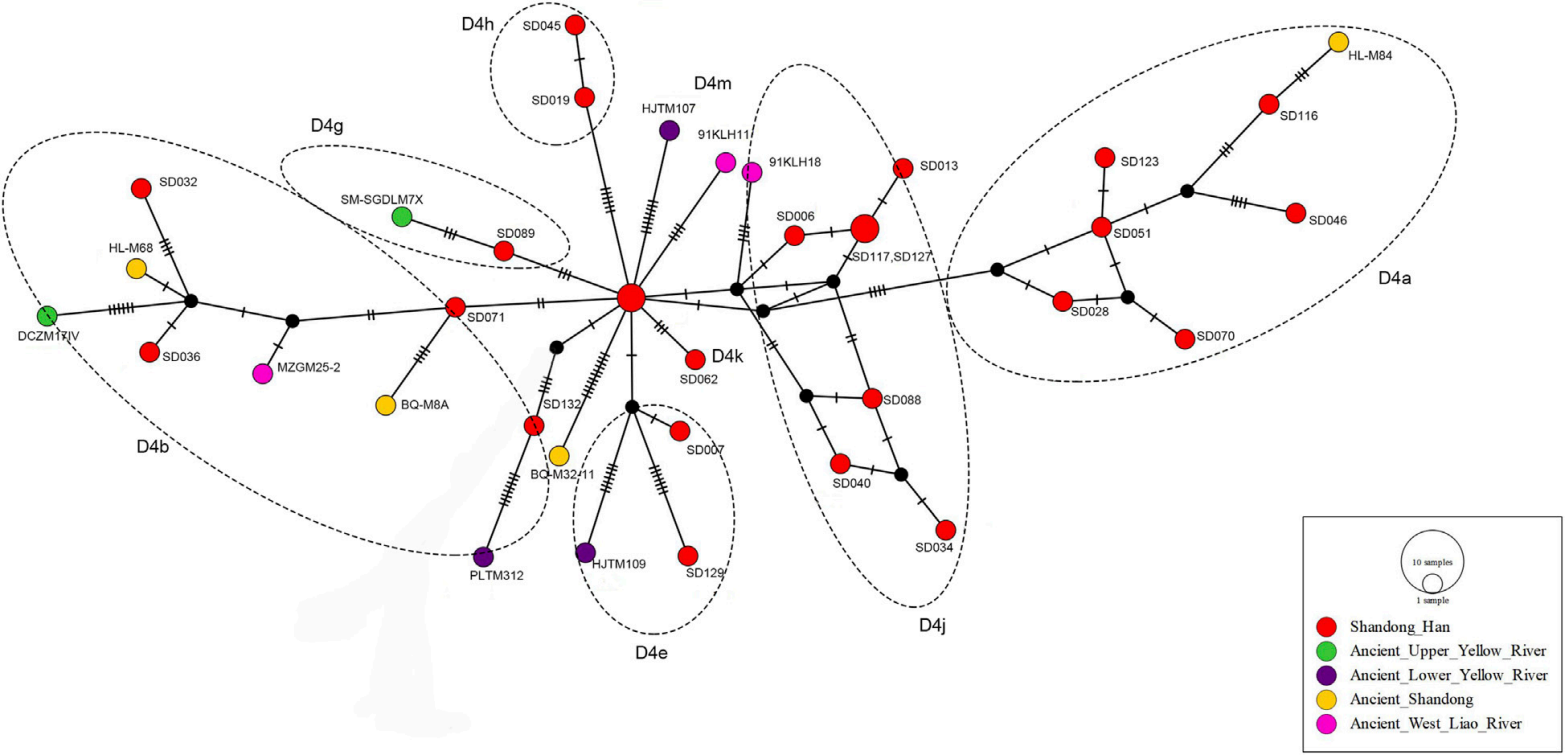
The median-joining network of haplogroup D4 appearing in modern Shandong Han coupled with reference populations from ancient Yellow River and West Liao River basins. The short black line is correlated with the number of different mutations, the shorter black line, the more different the mutations. The black internal node only represents data structure bifurcation points, rather than actual individuals.

To further explore the genetic background of the Shandong Han population, we analyzed the whole mitochondrial genomes of 36 ancient individuals from the Yellow River Basin, West Liao River Basin and Shandong, constructing genetic networks (Supplementary Table S3; Figure 3). In this study, haplogroup D4 was found at a high frequency (17.73%) in the Shandong Han population, with numerous downstream clades. Previous genome-wide studies of ancient populations from Northern East Asia, particularly those from the Yellow River Basin and the West Liao River Basin, have also identified haplogroup D4 as a prevalent type in these regions (Ning et al., 2020).

In recent research on maternal genetic structures, D4 was the dominant haplogroup in ancient Shandong populations between 9,500 and 1,800 years ago (Liu et al., 2021). As illustrated in Figure 3, we observed a strong genetic connection between the modern Shandong Han and ancient individuals from Shandong and the Lower Yellow River Basin, particularly within the sub-haplogroups D4a, D4b and D4e. Additionally, haplogroup D4j, which was common in ancient populations of the West Liao River Basin, was also present in modern Shandong Han. One Shandong Han individual belonging to sub-haplogroup D4g clustered with an ancient individual from Upper Yellow River Basin, with only three mutation difference.

Interestingly, sub-haplogroup D4h and D4k, which were not detected in the ancient populations, were discovered at the modern Shandong Han. This suggests that while the Shandong Han population shares significant genetic ties with ancient populations from the Yellow River and West Liao River Basins, their development likely involved the incorporation of maternal lineages from various sources.

**FIGURE 4:**
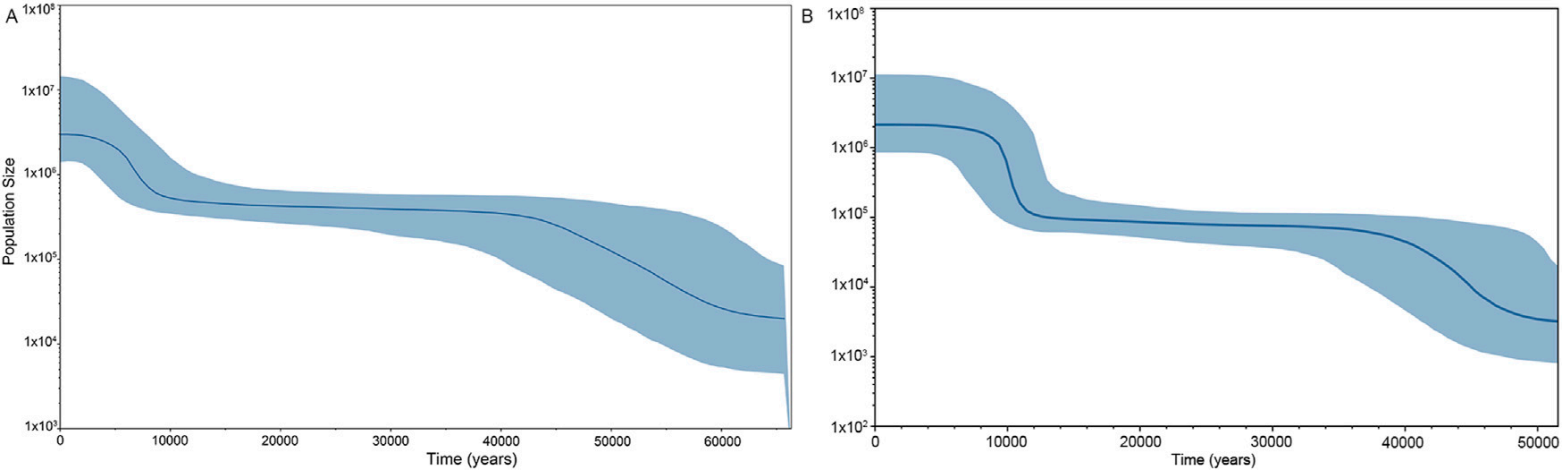
Bayesian skyline plot (BSP) showing change in effective population size of **(A)** Shandong Han based on the whole mitogenome and **(B)** Beijing Han of China (CHB). The *x*-axis and *y*-axis are representing the time and population size, receptively. The blue shaded area represents the 95% credibility interval whilst the lines represent posterior point estimators such as the median population size (solid blue line).

To assess the population expansion timeline of the Shandong Han population, we conducted a Bayesian skyline plot (BSP) analysis using whole mitochondrial genome data. As illustrated in Figure 4A, the BSP reflects the effective population size of the Shandong Han over time. The population underwent a significant expansion starting approximately 60,000 years ago (ka). This expansion peaked around 40,000 years ago and continued at a slower pace until about 10,000 years ago, when the population size reached equilibrium. A more recent growth phase occurred approximately 9,000 years ago, coinciding with the Neolithic period.

A similar pattern of population growth was observed in the CHB (Figure 4B), with a notable increase in population size around 10,000 years ago. This timeline aligns with the agricultural development seen in the Central Plains, as reported in previous studies (He et al., 2017; Robbeets et al., 2021). Research has shown that the adoption of agriculture by prehistoric societies played a key role in driving rapid population expansion (Gowdy, 2020).

These findings highlight the close connection between population growth in the Shandong Han and broader historical events, particularly the shift to agricultural practices that transformed human societies during the Neolithic era.

## 4 Conclusion

The study generated and submitted the whole mitochondrial genome data for 141 Han individuals from Shandong, Northern China, to the EMPOP dataset (accession number EMP00886). The results highlight that whole mitochondrial genome sequencing significantly improves genetic resolution and provides robust data for analyzing genetic diversity and other population metrics. The analysis of mtDNA haplogroups revealed that the majority of haplogroups in the Shandong Han population belong to East Asian lineages.

Population analyses further indicated that the Shandong Han not only share genetic links with ancient population from the Yellow River and West Liao River basins but have also been influenced by neighboring populations. Additionally, the Shandong Han experienced significant population expansion during the Neolithic period, aligning with similar growth patterns observed in the CHB population.

In conclusion, the mitochondrial genome data generated in this study will contribute to existing mitochondrial DNA databases in Northern China, providing deeper insights into the genetic composition of the Shandong Han. This dataset holds valuable potential for future archaeological and forensic applications.

## Data Availability

The datasets presented in this study can be found in online repositories. The names of the repository/repositories and accession number(s) can be found below: https://empop.online/populations, EMP00886.
